# Aspherical Surface Wavefront Testing Based on Multi-Directional Orthogonal Lateral Shearing Interferometry

**DOI:** 10.3390/s24237714

**Published:** 2024-12-02

**Authors:** Yahui Zhu, Ailing Tian, Hongjun Wang, Bingcai Liu

**Affiliations:** 1 School of Computer Science and Technology, Huaiyin Normal University, Huaian 223300, China; 2 School of Opto-Electronics Engineering, Xi’an Technological University, Xi’an 710021, China; ailintian@xatu.edu.cn (A.T.); whj0253@sina.com (H.W.); liubingcai@xatu.edu.cn (B.L.)

**Keywords:** aspherical surface wavefront testing, multi-directional orthogonal lateral shearing interferometry, measurement verification of wavefront testing

## Abstract

To overcome the limitations of phase sampling points in testing aspherical surface wavefronts using traditional interferometers, we propose a high-spatial-resolution method based on multi-directional orthogonal lateral shearing interferometry. In this study, we provide a detailed description of the methodology, which includes the theoretical foundations and experimental setup, along with the results from simulations and experiments. By establishing a relational model between the multi-directional differential wavefront and differential Zernike polynomials, we demonstrate high-spatial-resolution wavefront reconstruction using multi-directional orthogonal lateral shearing interferometry. Theoretical calculations and simulations of aspherical surface wavefront testing are followed by experimental verification on an aspherical surface with a known asphericity. Comparing the measurement results with those from the LuphoScan profilometer, we achieve a relative measurement error with an RMS precision better than λ/100.

## 1. Introduction

There are specific technical challenges in measuring the wavefront of aspheric surfaces. For example, the complex shape of aspheric mirrors can lead to limitations in traditional wavefront testing methods (such as interferometry), including a low resolution, large measurement errors, or strict test condition requirements. The application of traditional lateral shearing interferometers in aspheric surface testing has several limitations [1,2]:

1. Limited shearing amount: Traditional interferometers may not have enough shearing, leading to unclear interference fringes and inaccurate wavefront measurements.
2. Measurement accuracy: In cases of large wavefront distortion, traditional methods may fail to provide accurate results.
3. Vulnerability to environmental factors: The stability of traditional equipment may be insufficient in complex testing environments.
4. Operational complexity: Traditional interferometers require complex adjustments and optimization, making them unsuitable for large-scale or high-precision automated testing.

Traditional lateral shearing interferometers are useful for a qualitative analysis of aberrations, but quantitative wavefront demodulation requires collecting interferograms in two shear directions to calculate the wavefront distribution. This process hinders real-time wavefront testing and introduces additional angle errors due to changes in the shearing direction. Multi-directional wavefront shearing interferometry uses lateral shearing interference from multiple directions to obtain wavefront shearing information at different angles. To date, a rotatable shearing interferometer has been developed based on orthogonal polarization theory, demonstrating excellent spatial suppression and a high testing accuracy [3]. A lateral wavefront shearing interferometer that uses two diffraction gratings for beam splitting can change the shearing direction by rotating the gratings in opposite directions [4]. For example, a variable lateral shearing interferometer using two holographic cross diffraction gratings was proposed for testing non-rotational symmetric wavefronts with aberrations greater than 100λ (λ is wavelength) and slope changes greater than 40λ/diameter [5]. By using a differential rotational shearing interferometer to test deviations in rotating asymmetric aspherical surfaces, the sensitivity increases as the shear angle decreases, and the number of fringe patterns displayed on the pupil can be controlled by the shear angle [6]. A vector shearing interferometry device was proposed [7,8,9], which allows adjustable shear and tilt in any direction by selecting orthogonal displacements. The number and direction of interference fringes can be controlled by the shear direction and its magnitude. To address the impact of arbitrary shear direction and variable shear offset on wavefront estimation, a wavefront recovery method was proposed [10], which uses a regularization technique to estimate the wavefront from vector shear interferograms with variable amplitude and shear direction. By analyzing the differences in wavefronts sheared in multiple directions, the relationship between adjacent pixels is established. The differential wavefronts from multiple shear directions are then analyzed, and through time modulation of the interferograms at each angle, the local wavefront gradient is obtained, enabling high-resolution wavefront reconstruction [11,12,13]. To address the limitations of traditional interferometers, and with the development of multi-directional shearing technology, this paper proposes a multi-directional orthogonal lateral shearing interferometry. Overall, its advantages include the following: multi-directional shearing, which provides more interference fringe data and reduces errors from single-direction shearing; orthogonal shearing, which enhances system fault tolerance and improves fringe clarity, thus boosting wavefront measurement accuracy; and, in terms of precision, this advanced technique delivers higher accuracy for complex wavefronts (e.g., aspherical surfaces), especially in testing optical components with intricate shapes.

The fringe density in the interference field of a lateral shearing interferometry system depends not only on the measured wavefront but also on the lateral offset between wavefronts. Therefore, by controlling the lateral offset on the detector plane, the fringe densification caused by non-null aspherical aberrations must also be effectively suppressed. This is the mechanism for achieving non-null aspherical wavefront testing in lateral shearing interferometry systems. In recent years, Y Yang et al. [14] proposed a testing technique that combines shearing interferometry with partial compensation to measure deep aspherical surfaces. H Wang et al. [15] used the phase-shifting lateral shearing interferometry for aspherical surface measurement experiments, where the RMS relative error was optimized under varying amounts of shearing error. Additionally, radial shearing interferometry was used to verify aspherical testing technology, as shown in Ref. [16], among others. The existing problems are quite evident, including the slow development and low accuracy associated with using lateral shearing interferometry for measuring aspherical surfaces. Recently, Zhejiang University replaced the interference device with a quadria-wave lateral shearing (achieving lateral shearing in two directions simultaneously) interferometry system [17]. They combined a part-null compensator (PNC) with the quadria-wave lateral shearing interferometer (QWLSI) for testing large-curvature and highly aspherical surfaces. Additionally, the design principle of a lateral shearing interferometry system using a randomly encoded hybrid grating (REHG) was proposed [17,18], and REHG was applied to create a universal testing system for non-null aspherical surfaces. Theoretical simulations showed that the RMS value of surface reconstruction under ideal conditions is about 5 × 10^−3^λ. Meanwhile, quadria-wave lateral shearing interference was used for calculation and simulation to achieve aspherical wavefront testing, demonstrating that the fitting error precision reaches the order of 10^−13^ [19]. In summary, shearing interferometry has become a key research focus in aspherical wavefront testing. In lateral shearing interferometry, wavefront surface information can only be reconstructed by obtaining interferograms in two orthogonal directions. However, using interferograms with just one orthogonal shear direction poses problems, such as the low precision in surface wavefront reconstruction due to insufficient sampling points and the presence of rotational and random errors.

The main focus of our research is the use of multi-directional shearing polarization interferometry to modulate multiple fixed orthogonal shearing interferograms, thereby obtaining more phase information for high-precision reconstruction of aspherical wavefronts. This paper proposes a multi-directional orthogonal lateral shearing interferometry method for testing aspherical surface wavefronts, aiming to improve the spatial wavefront information in shearing interferograms. The technique uses polarized birefringent crystals for orthogonal shearing in the interferometry system, and a fixed orthogonal shear model (orthogonal shearing beam displacer) is developed. A multi-directional orthogonal lateral shearing interferometry system is also designed to estimate wavefronts using multi-directional orthogonal phase sampling points.

In this paper, we propose a theory and implementation method of aspherical surface wavefront testing based on multi-directional orthogonal lateral shearing interferometry. Section 2 presents the methodology of our investigations, providing a detailed description of the proposed method, including its theoretical foundations and experimental setup. Section 3 presents results, which are discussed separately for simulations and experiments. Section 4 analyzes the results, comparing them with existing methods and addressing the implications of some testing errors. Section 5 concludes the paper, summarizes the findings, and suggests directions for future work.

## 2. Methodology

### 2.1. The Principle of Multi-Directional Orthogonal Lateral Shearing Interferometry

**Figure 1:**
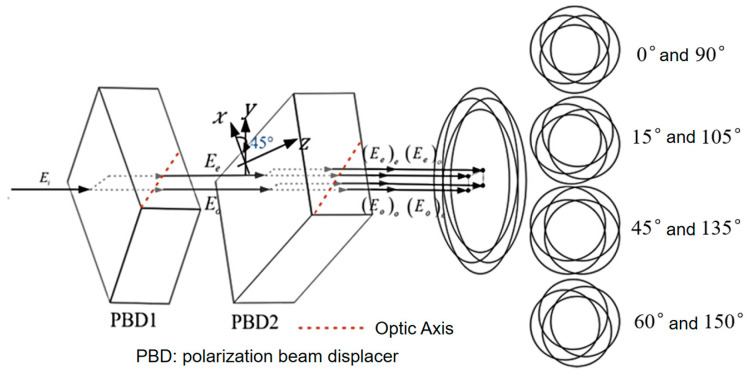
The principal diagram of multi-directional orthogonal lateral shearing interferometry (The arrow represents the light propagation direction).

To obtain two sets of orthogonal lateral shearing interferograms from a single image, a multi-directional orthogonal lateral shearing interferometry scheme was designed. The basic principle of wavefront testing using orthogonal lateral shearing interferometry is shown in Figure 1. Figure 1 illustrates the principle of multi-directional orthogonal lateral shearing interferometry. Its optical configuration consists of two polarization beam splitters (PBD1 and PBD2). The fast axis of PBD1 is aligned along the *x*-axis, while the fast axis of PBD2 is rotated 45° relative to the *y*-axis. These two birefringent crystals form a double birefringent crystal beam displacer (DBCs-BD) system that creates an orthogonal shearing displacer. Four orthogonally polarized overlapping light waves are emitted from the DBCs-BD system. When passing through an analyzer with a 45° or 135° transmission polarization azimuth, a single frame of the Charge-Coupled Device (CCD) captures one interferogram in any direction, containing two sets of fixed orthogonal wavefronts.

After the lateral shearing light waves pass through PBD2, the ordinary (*o*)-light and extraordinary (*e*)-light are separated into two sheared light waves in the y-direction. These two sets of light waves are then processed by the DBCs-BD orthogonal lateral shearing system, forming four linearly polarized light waves with orthogonal shear. These waves are represented as $E_{1}={\left(E_{o}\right)}_{o}$, $E_{2}={\left(E_{o}\right)}_{e}$, $E_{3}={\left(E_{e}\right)}_{o}$ and $E_{4}={\left(E_{e}\right)}_{e}$, respectively. The four replicated light waves generated by the DBCs-BD system contain two sets of orthogonal overlapping polarized light beams. The polarization vectors of these four light waves are represented as $E_{x-}\left(x+s,y\right)=E_{1}=E_{\left(o\right)o}$, $E_{x+}\left(x-s,y\right)=E_{3}=E_{\left(e\right)o}$, $E_{y+}\left(x,y-s\right)=E_{2}=E_{\left(o\right)e}$, $E_{y-}\left(x,y+s\right)=E_{4}=E_{\left(e\right)e}$, respectively.

The four linearly polarized light waves emitted from the DBCs-BD system, which overlap, are incident on the analyzer. When the analyzer’s polarization angle is set to 45° or 135°, the complex amplitudes of the four sheared wavefronts are given by the following:

(1) $$E_{x-}\left(x,y\right)=\frac{A}{2}\cos\theta \left[\begin{array}{c} \cos\alpha \\ -\sin\alpha \end{array}\right]\exp\left[j\left(W\left(x+s,y\right)-2\pi f_{0}x\right)\right],$$

(2) $$E_{x+}\left(x,y\right)=\frac{A}{2}\sin\theta \left[\begin{array}{c} \sin\alpha \\ \cos\alpha \end{array}\right]\exp\left[j\left(W\left(x-s,y\right)+2\pi f_{0}x\right)\right],$$

(3) $$E_{y-}\left(x,y\right)=\frac{A}{2}\cos\theta \left[\begin{array}{c} \cos\alpha \\ -\sin\alpha \end{array}\right]\exp\left[j\left(W\left(x,y+s\right)-2\pi f_{0}y\right)\right],$$

(4) $$E_{y+}\left(x,y\right)=\frac{A}{2}\sin\theta \left[\begin{array}{c} \sin\alpha \\ \cos\alpha \end{array}\right]\exp\left[j\left(W\left(x,y-s\right)+2\pi f_{0}y\right)\right].$$

The above Equations (1)–(4) are referenced (reprinted with permission from Ref. [20], Equations (5)–(8). © 2024 Optical Society of America): W(x,y) presents the wavefront under test, θ presents the incident angle of light wave $E_{i}$, and j presents the phase difference between two optical fields. The rotation angle of PBD2 is denoted by α, and $\alpha =\omega \left(n_{o}-n_{e}\right)Z/2c$, the $n_{o}$, $n_{e}$ present the refractive index of the *o*-light and *e*-light respectively, the ωZ/2c is the resonance ratio, and *ω* is the angular frequency, The term *Z*/2*c* represents the correlation coefficient of polarization characteristics, while s denotes the shear offset distance (displacement amount), and $f_{0}$ represents the spatial frequency. These four overlapping replicated light waves, after passing through the analyzer, undergo polarization orthogonal lateral shearing interferometry. This effect arises from the orthogonal superposition of the four light waves $E_{x+}$, $E_{x-}$, $E_{y+}$ and $E_{y-}$, resulting in polarization interference.

**Figure 2:**
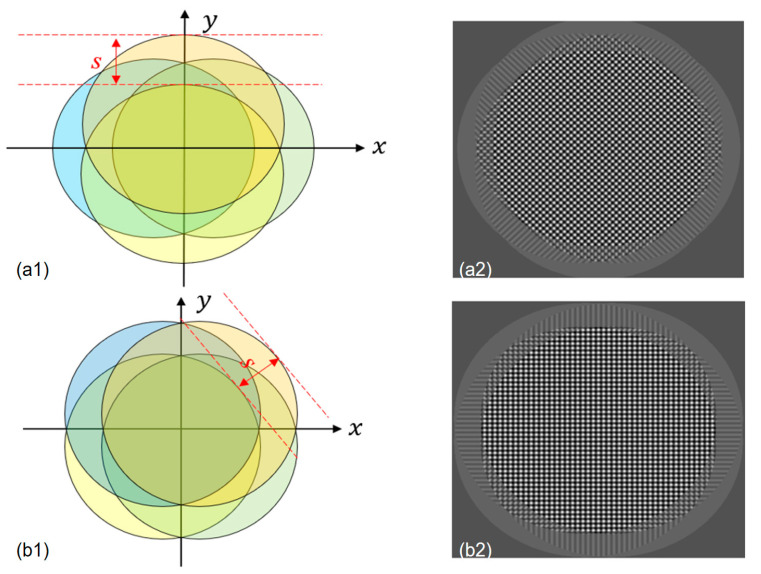
Fringe feature distribution in orthogonal lateral shearing interferometry. (**a1**) Wavefront position distribution map in orthogonal lateral shearing interferometry. (**a2**) Orthogonal shearing fringe pattern corresponding to (**a1**). (**b1**) Wavefront spatial distribution diagram with the DBC-BD system rotated 45° around the *z*-axis. (**b2**) Orthogonal shearing fringe pattern corresponding to (b1).

The fringes pattern distribution of multi-directional orthogonal lateral shearing interferometry is shown in Figure 2. This technique allows for lateral shearing interferometry in two orthogonal directions simultaneously, enabling the measurement of the wavefront’s spatial position distribution across four polarization components detected at the imaging plane. However, shearing interferometry is used to measure the deviation of the wavefront under test. By analyzing the interferometric fringes on an aspherical surface, the wavefront deviation of the surface can be obtained. For non-rotationally symmetric wavefronts, lateral shearing interferometry requires two fringe patterns. Due to the complexity of the fringes on the aspherical surface, wavefront reconstruction is necessary to determine the aberrations caused by the surface. High-precision testing with lateral shearing interferometry requires precise knowledge of both the shear amount and shear direction. It cannot achieve high-precision wavefront testing with large deviations. Therefore, this method is most effective for testing aspherical surfaces with small deviations.

Assuming that the wavefront under test, after incident on the DBC-BD system, has a distribution, the shearing wavefronts in the x- and y-directions can be represented as follows:

(5) $$\Delta W_{x}(x,y)=W(x-s,y)-W(x+s,y)$$

(6) $$\Delta W_{y}(x,y)=W(x,y-s)-W(x,y+s)$$

In this context, ΔW represents the value to be measured in lateral shearing interferometry. The optical path difference ΔW at each point on wavefront of the aspherical surface can be expressed by the commonly used relationship. When the shear amount s is small and the shear directions are orthogonal along the *x*- and *y*-axes, the following expression can be obtained as follows:

(7) $$\Delta W_{x}(x,y)=\frac{\partial W_{x}(x,y)}{\partial x}\cdot s=N\lambda$$

(8) $$\Delta W_{y}(x,y)=\frac{\partial W_{y}(x,y)}{\partial y}\cdot s=N\lambda$$

In this case, *N* represents the order of the aspherical wavefront interferometry fringes.

W(x,y) is calculated from the orthogonal lateral shearing interferogram, and, by separately integrating the above equation, the aberration of the wavefront under test can be determined as follows:

(9) $$W_{x}(x,y)=\frac{1}{s}\int \Delta W_{x}(x,y)dx+c$$

(10) $$W_{y}(x,y)=\frac{1}{s}\int \Delta W_{y}(x,y)dy+c$$

The shearing interferogram is a derivative of the wavefront under test in the shear direction. Therefore, it is necessary to recover the original wavefront from the obtained interferograms. Based on the expression for aspherical wavefront aberration, shearing interferograms with various primary aberrations can be roughly determined.

### 2.2. Implementation Methods for Wavefront Reconstruction

When obtaining any two sets of orthogonal lateral shearing wavefronts in different directions, the original wavefront is represented by the first *N*-terms of the Zernike polynomials as follows:

(11) $$W(i,i+90^{\circ })=\sum_{j=1}^{N}a_{j}Z_{j}(i,i+90^{\circ })$$

In this expression, $W(i,i+90^{\circ })$ represents differential wavefront in any orthogonal direction, $a_{j}$ represents coefficient, and $Z_{j}(i,i+90^{\circ })$ represents Zernike polynomial. The result derived by using Equation (11) shows that the two orthogonal shearing wavefronts $\Delta W_{i}(i,i+90^{\circ })$ and $\Delta W_{i+90^{\circ }}(i,i+90^{\circ })$ are represented as follows:

(12) $$\begin{array}{ll} \Delta W_{i}(i,i+90^{\circ }) & =W(i-s,i+90^{\circ })-W(i+s,i+90^{\circ })\\ & =\sum_{j=1}^{N}a_{j}[Z_{j}(i-s,i+90^{\circ })+Z_{j}(i+s,i+90^{\circ })] \end{array}$$

(13) $$\begin{array}{ll} \Delta W_{i+90^{o}}(i,i+90^{\circ }) & =W(i,(i+90^{\circ })-s)-W(x,(i+90^{\circ })+s)\\ & =\sum_{j=1}^{N}a_{j}[Z_{j}(i,(i+90^{\circ })-s)+Z_{j}(i,(i+90^{\circ })+s)] \end{array}$$

Assuming that the differential Zernike polynomials for any two different fixed orthogonal directions are given by the following:

(14) $$\Delta Z_{i}=Z_{j}(i-s,i+90^{\circ })+Z_{j}(i+s,i+90^{\circ })$$

(15) $$\Delta Z_{i+90^{\circ }}=Z_{j}(i,(i+90^{\circ })-s)+Z_{j}(i,(i+90^{\circ })+s)$$

By establishing the corresponding relationship between the differential wavefront and the differential Zernike polynomial basis functions, as given in Equations (12) to (15) above, the following results are obtained as follows:

(16) $$\Delta W_{i}(i,i+90^{\circ })=\sum_{j=1}^{N}a_{j}\Delta Z_{i}$$

(17) $$\Delta W_{i+90^{\circ }}(i,i+90^{\circ })=\sum_{j=1}^{N}a_{j}\Delta Z_{i+90^{\circ }}$$

The two equations above can be represented in matrix form as follows:

(18) $$\Delta W=\Delta Za_{i,i+90^{\circ }}$$

Among them, the matrix representations of the differential wavefront and the differential Zernike polynomial basis function are as follows:

(19) $$\Delta W=\left(\begin{array}{c} \Delta W_{i}\\ \Delta W_{i+90^{\circ }} \end{array}\right),\;\Delta Z=\left(\begin{array}{c} \Delta Z_{i}\\ \Delta Z_{i+90^{\circ }} \end{array}\right)$$

Expand the row dimension of the matrix in Equation (19) as follows. The matrix expansions for the differential wavefront and the differential Zernike polynomials are as follows:

(20) $$\Delta W=\left(\begin{array}{c} \Delta W_{i}\\ \begin{array}{c} \Delta W_{i+90^{\circ }}\\ \Delta W_{i+1} \end{array}\\ \Delta W_{\left(i+1\right)+90^{\circ }}\\ \begin{array}{c} \vdots \\ \begin{array}{c} \Delta W_{i+N}\\ \Delta W_{\left(i+N\right)+90^{\circ }} \end{array} \end{array} \end{array}\right),(i=0,1,2,\cdots ,j,N=j+1),\Delta Z=\left(\begin{array}{c} \Delta Z_{i}\\ \Delta Z_{i+90^{\circ }}\\ \begin{array}{c} \Delta Z_{i+1}\\ \Delta Z_{\left(i+1\right)+90^{\circ }} \end{array}\\ \begin{array}{c} \vdots \\ \begin{array}{c} \Delta Z_{i+N}\\ \Delta Z_{\left(i+N\right)+90^{\circ }} \end{array} \end{array} \end{array}\right),(i=0,1,2,\cdots ,j,N=j+1)$$

The coefficients of the Zernike polynomial, obtained by solving the least squares of Equation (18), are as follows:

(21) $$a_{i,i+90^{\circ }}={\left(\Delta Z^{T}\Delta Z\right)}^{-1}\Delta Z^{T}\Delta W,i=0,1,2,\cdots ,j$$

By taking the average of the N sets of differential wavefronts, calculate the coefficients of the N sets of Zernike polynomials, sum all the coefficients, and then take the average again to obtain the value of the fitting coefficients:

(22) $$a=\frac{1}{N}\sum_{i=0^{\circ }}^{j}a_{i,i+90^{\circ }},i=0,1,2,\cdots ,j;\;N=j+1.$$

Finally, by substituting the coefficients of the Zernike polynomial into Equation (11), the wavefront under test is reconstructed:

(23) $$W(i,i+90^{\circ })=\sum_{j=1}^{N}a_{j}Z_{j}(i,i+90^{\circ }),\;(i=0,1,2,\cdots ,j,N=j+1)$$

Multi-sets of mutually orthogonal differential wavefront phase distributions are obtained by precisely rotating through an orthogonal shearing system. These differential phases are then averaged across the multi-sets, and the coefficients of the differential Zernike polynomials are calculated by fitting the differential Zernike polynomials to the data. The corresponding basis function coefficients of the differential Zernike polynomials for the multi-sets of orthogonal differential phases are obtained. Finally, by averaging the basis function coefficients from different orthogonal directions, the wavefront under test is reconstructed using these coefficients.

### 2.3. Theoretical Foundations for Testing Aspherical Surfaces

**Figure 3:**
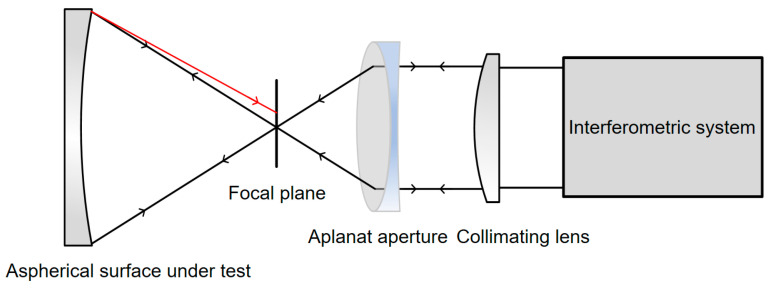
Schematic diagram of retrace error generated by aspherical surface testing.

In aspherical surface testing, due to wavefront deviations, light reflected from the surface cannot converge to a point. As a result, the incident light wave on the parabolical surface does not retrace its original path, but instead reflects to a point on the focal plane, which it to forming a diffuse spot. This is known as the retrace error caused by asphericity, as shown in Figure 3.

In actual testing of aspherical surfaces, parallel light emitted by the interferometric system converges to its focal point through an aplanatic aperture, generating a spherical wavefront. This spherical wavefront then reaches the aspherical surface, where it is reflected back to the interferometric system. The system directly measures the deviation of the aspherical surface from the spherical reference wavefront.

**Figure 4:**
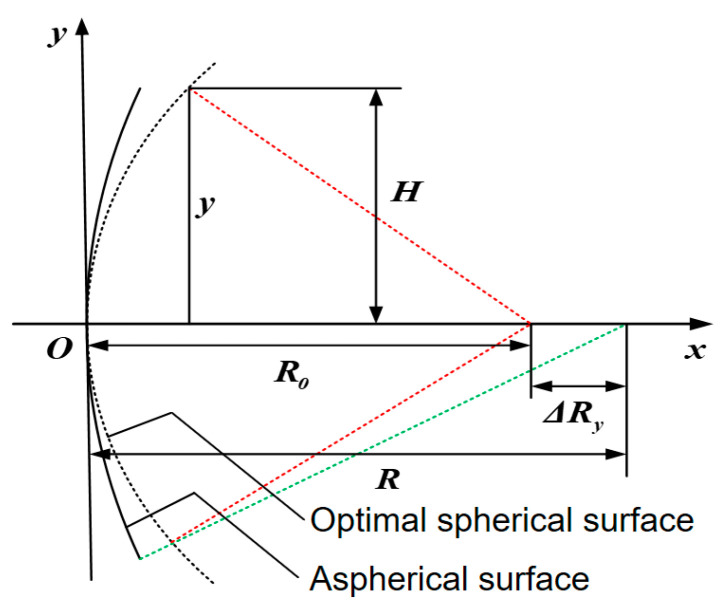
Schematic diagram of the optimal spherical surface for concave aspherical surfaces (The red dotted line represents incident light and green dotted line represents reflected light).

To obtain accurate measurements, it is necessary to determine the comparison spherical surface that is closest to the aspherical surface, known as the optimal spherical surface. Figure 4 shows a schematic diagram of the optimal spherical surface for concave aspherical surfaces.

By taking the curvature radius of the aspherical vertex as $R_{0}$, the eccentricity as *e*, and aspherical aperture as *D* (with *D* = 2*H*) as known conditions, the radius R for the optimal spherical surface can be determined using an analytical method. Taking a quadratic aspherical paraboloid as an example, the equation for the paraboloid is expressed as follows:

(24) $$y^{2}=2R_{0}x-(1-e^{2})x^{2}$$

The intersection points between the aspherical normal and the optical axis vary with the height of different zones, as shown in Figure 4. Assuming that the distance from the intersection point of the paraxial normal and the optical axis to the aspherical vertex is $R_{0}$, and the distance from the intersection point of the optical axis to the aspherical vertex in the y-direction is $R_{y}$, the difference in normal intercept is expressed as follows:

(25) $$\Delta R_{y}=R-R_{0}=e^{2}x=e^{2}\frac{R_{0}-\sqrt{{R_{0}}^{2}-(1-e^{2})y^{2}}}{1-e^{2}}$$

From Equation (25), it can be seen that for a parabolic surface, $\Delta R_{y}=x$, on the side, and for a spherical surface, $\Delta R_{y}=0$, it is expressed as follows:

(26) $$x_{sphere}=R-\sqrt{R^{2}-H^{2}}=R-R\sqrt{1-{\left(\frac{H}{R}\right)}^{2}}$$

Expand Equation (26) using a series to the following:

(27) $$x_{sphere}=\frac{1}{2}\cdot \frac{H^{2}}{R}+\frac{1}{8}\cdot \frac{H^{4}}{R^{3}}+\frac{1}{16}\cdot \frac{H^{6}}{R^{5}}+\frac{1}{128}\cdot \frac{H^{8}}{R^{7}}+\ldots$$

For aspherical surfaces, the following can also be obtained:

(28) $$x_{aspheric}=\frac{1}{2}\cdot \frac{H^{2}}{R_{0}}+\frac{\left(1-e^{2}\right)}{8}\cdot \frac{H^{4}}{{R_{0}}^{3}}+\frac{{\left(1-e^{2}\right)}^{2}}{16}\cdot \frac{H^{6}}{{R_{0}}^{5}}+\frac{{\left(1-e^{2}\right)}^{3}}{128}\cdot \frac{H^{8}}{{R_{0}}^{7}}+\ldots$$

The closest comparison spherical surface is at the height of y=D/2=H, which should be $x_{sphere}=x_{aspheric}$. Take the first two terms of the series, respectively:

(29) $$\frac{1}{2}\cdot \frac{H^{2}}{R}+\frac{1}{8}\cdot \frac{H^{4}}{R^{3}}\approx \frac{1}{2}\cdot \frac{H^{2}}{R_{0}}+\frac{\left(1-e^{2}\right)}{8}\cdot \frac{H^{4}}{{R_{0}}^{3}}$$

By omitting high-order small quantities, one can obtain the following:

(30) $$R\approx \frac{R_{0}}{1-\frac{e^{2}A^{2}}{64}}$$

In Equation (30), *A* represents the relative aperture of the aspherical surface.

The eccentricity of a paraboloid is *e =* 1, and, by substituting this into Equation (30), the radius of the optimal spherical surface for the paraboloid is obtained as follows:

(31) $$R=R_{0}+\frac{A\cdot D}{32}=R_{0}+\frac{H^{2}}{4R_{0}}$$

**Figure 5:**
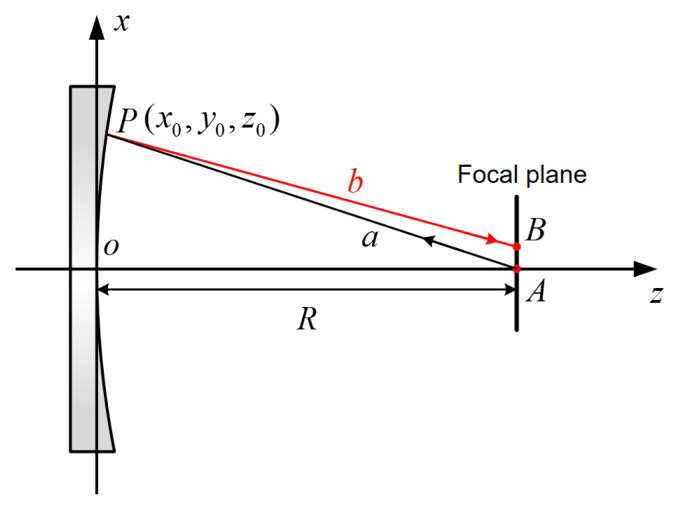
Schematic diagram of the ray tracing for solving the deviation of aspherical surfaces.

From the above, the radius of the optimal spherical surface (a sphere selected to closely match the measured wavefront by minimizing errors or deviations) is determined. This radius is used to calculate the distance between the aspherical surface and the spherical standard mirror in actual experiments. The results directly measured through the experimental system represent the deviation of the aspherical surface under test from the optimal spherical surface. To obtain the deviation of the aspherical surface from its design value, a corresponding optical path model must be established based on the actual testing setup. The ray-tracing method is then used to determine the deviation between the ideal aspherical surface and the optimal spherical surface. The optimal spherical surface is subsequently removed from both the tested and calculated values, allowing the wavefront deviation between the aspherical surface under test and the ideal aspherical surface to be obtained. A schematic diagram of the ray-tracing method used to solve the deviation between the ideal aspherical surface and the optimal spherical surface is shown in Figure 5.

In this optical path model, the beam from the spherical standard mirror converges to point *A* and then diverges, generating a spherical wavefront that partially compensates for the paraboloid under test. The incident light, denoted as *AP*, interacts with the surface. However, since the partially compensated spherical wavefront cannot fully compensate for the phase difference along the normal to the paraboloid, the testing wavefront incident on the paraboloid cannot retrace its original path. The reflected light is denoted as *PB*. Due to the non-common path between the incident light and the reflected light, the optical path difference (*OPD*) changes, resulting in a retrace error caused by asphericity. This error represents the deviation introduced by the aspherical surface.

(32) $$OPD=\left|\vec{AP}\right|-\left|\vec{PB}\right|$$

Let the coordinates of point *P* be $\left(x_{0},y_{0},z_{0}\right)$. The distance from the focal point of an aplanatic aperture to the center of the aspherical surface is its closest curvature radius to the spherical surface, denoted as *R*. The incident light is represented by $\vec{AP}=(x_{0},y_{0},z_{0}-R)$, with the following assumption:

(33) $$\left|\vec{AP}\right|=\sqrt{{x_{0}}^{2}+{y_{0}}^{2}+{\left(z_{0}-R\right)}^{2}}$$

According to the theory of spatial ray tracing and the law of reflection, the spatial coordinates of point *B* can be obtained for the reflected light path, *PB*. Let the equation of the aspherical surface under test be defined as follows:

(34) $$z=\frac{c\cdot (x^{2}+y^{2})}{1+\sqrt{1-(1+k)c^{2}(x^{2}+y^{2})}}$$

In this context, *c* represents curvature, and *k* denotes the quadratic conic coefficient of aspherical surfaces. The normal vector at point *P* is determined by taking the partial derivatives of the aspherical surface equation. Take the partial derivatives of aspherical equation to obtain the normal vector. According to the principle of light reflection in spatial, the Cartesian coordinate component of the unit direction vector *b* of the reflected light at point *P* are expressed as follows:

(35) $$\left\{ \begin{array}{l} b_{x}=a_{x}-2n_{x}a\cdot n\\ b_{y}=a_{y}-2n_{y}a\cdot n\\ b_{z}=a_{z}-2n_{z}a\cdot n \end{array}\right.$$

In which, a represents the unit direction vector of the incident light, and *n* denotes the normal vector. The reflected light vector *PB* is given by the following expression:

(36) $$\frac{x-x_{0}}{b_{x}}=\frac{y-y_{0}}{b_{y}}=\frac{z-z_{0}}{b_{z}}$$

Therefore, the *OPD* is expressed as follows:

(37) $$OPD=\left|\vec{AP}\right|-\left|\vec{PB}\right|=\sqrt{\frac{{b_{x}}^{2}(z_{0}+r)}{b_{z}}+\frac{{b_{y}}^{2}(z_{0}+R)}{b_{z}}+(R-z_{0})}-\sqrt{{x_{0}}^{2}+{y_{0}}^{2}{\left(z_{0}-R\right)}^{2}}$$

The *OPD* calculated using the ray-tracing method result in defocusing due to the difficulty in accurately determining the *OA* distance. In theory, the *OPD* is the sum of the retrace error and the defocus caused by the deviation of the ideal aspherical surface from the optimal spherical surface. Therefore, the retrace error caused by asphericity can be obtained by subtracting the defocus from the OPD. Therefore, Zernike polynomials can be used to fit the *OPD*. To model this, Zernike polynomials can be used to fit the OPD. Due to their orthogonality on normalized unit circles, Zernike polynomials provide a good correspondence with optical aberrations. Thus, any wavefront deviation can be expressed using Zernike polynomials as follows:

(38) $$OPD=\sum_{i=0}^{n}a_{i}\cdot Z_{i}(\rho ,\theta )$$

In which, ρ represents the radial distance, θ represents the angle, and *n* represents the fitting order. Based on the above theoretical analysis, it is obtained that $OPD=E_{Surface}+E_{Power-Error}$, where the $E_{Power-Error}$ represents the defocusing error. Therefore, by combining the Zernike polynomial, the expression for the wavefront deviation $E_{Surface}$ of the ideal parabolic surface relative to the optimal spherical surface is expressed as follows:

(39) $$E_{Surface}=\sum_{i=0}^{\infty }a_{i}Z_{i}(\rho ,\theta )-E_{Power-Error}$$

The wavefront deviation of the ideal aspherical surface relative to the optimal spherical surface can be obtained from Equation (39). By subtracting the calculated value of the ideal aspherical surface from both the actual measured value and the optimal spherical surface, the surface deviation between the aspherical surface under test and the ideal aspherical surface is determined.

### 2.4. Experimental Setup

**Figure 6:**
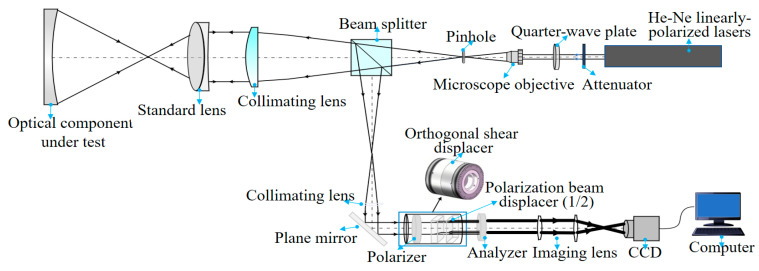
Optical path diagram of multi-directional orthogonal lateral shearing interferometry.

The experiment was based on multi-directional orthogonal lateral shearing interferometry. The optical setup for aspherical wavefront testing is shown in Figure 6. As seen in the figure, the light emitted by the He-Ne laser is first intensity-modulated using an attenuation plate, then converted into circularly polarized light by a quarter-wave plate. During the experiment, the orthogonal shear displacer must be rotated to prevent it from affecting the polarization state. The displacer consists of two birefringent crystals, with a polarization beam displacer (PBD) splitting the test beam into orthogonal *o*-beams and *e*-beams to generate lateral shear. The PBD1 shears the two beams laterally, and PBD2 modulates them into four sets of linearly polarized beams in orthogonal directions. Since these beams do not create interference patterns on their own, they must pass through a polarizer set at 45° or 135° to generate interference. The four beams then interfere in the orthogonal direction, and after being analyzed by a polarizer, an orthogonal lateral shearing interference pattern is produced. This multi-directional shear interferogram is captured by a CCD camera.

**Figure 7:**
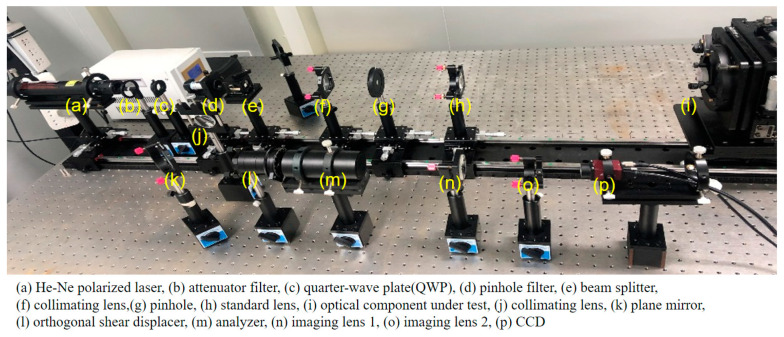
Experimental setup of multi-directional orthogonal lateral shearing interferometry.

The experimental system was constructed based on the structural principle of multi-directional orthogonal lateral shearing interferometry, and the experimental setup is shown in Figure 7. The setup includes the following components: The interferometer consists of the optical component under test, a standard lens (convex lens, aperture: 50.8 mm, focal length: 200 mm, @Thorlabs Inc. Newton, NJ, USA), a collimating lens (when a collimating lens and a standard lens are used together, methods such as wavefront analysis and optimization design are employed to minimize deviations in the reference wavefront), a beam splitter, a linear polarizer, two birefringent crystals (PBD1 and PBD2), an analyzer (a linear polarizer, LPVIS100, extinction ratio: 800:1, @Thorlabs Inc.), an imaging lens (LB1596-A, diameter: Ø = 25.4 mm, focal length: 150 mm, @Thorlabs Inc.), and a CCD camera (2/3 inch Sony CMOS Pregius sensor, IMX250, resolution: 2448 × 2048, 5 MP, 75 fps, @The Imaging Source, Inc. Berlin, Germany). These components are arranged coaxially along the same optical axis. The birefringent crystals (calcite, polarization parallel plate, CN2103091401, @Union Optic Inc. Wuhan, China) are 9.28 mm thick and serve as the PBDs for the orthogonal shearing of the wave in the x- and y-directions. The lateral shearing distance introduced by the PBD is 1 mm, and the ordinary and extraordinary waves are laterally shifted by 1/10 of the incident beam aperture in the *x*- or *y*-axis direction. In addition, the interferometer includes a laser light source (HRS015B, He-Ne laser, max power: <5 mW, wavelength: 632.99 nm, linearly polarized, @Thorlabs Inc.), an attenuator filter (GCO-07 series circular adjustable attenuator, @Thorlabs Inc.), a microscope objective (magnification: ×40, NA: 0.65, working distance: 0.632 mm, focal length: 4.65 mm, field of view (FN): 0.5 mm, @Thorlabs Inc.), and a pinhole (GCO-P05A, pinhole diameter: 5 μm, seat diameter: Ø 25.4 mm, @DHC Inc., Tokyo, Japan), which, together, form the beam-expanding collimation system. These components are arranged parallel to and concentric with the main optical axis. PBD1 is oriented horizontally along the *x*-axis, with its fast axis aligned at 0° to the *x*-axis. PBD2 is rotated 45° along the *y*-axis relative to PBD1. The orthogonal shearing waves produced by the PBDs are laterally sheared in the orthogonal direction and undergo interference when passing through a 45° or 135° analyzer.

## 3. Results

### 3.1. Simulation Results

#### 3.1.1. Wavefront Reconstruction

Using the aspherical formula to subtract the spherical surface in order to generate an aspherical wavefront, according to the general expression for an aspherical surface given by Equation (34), the resulting expression is as follows:

(40) $$W=\frac{c\cdot \left(x^{2}+y^{2}\right)}{1+\sqrt{1-(k+1)c^{2}(x^{2}+y^{2})}}$$

(41) $$W=W_{aspheric}-W_{sphere}$$

**Figure 8:**
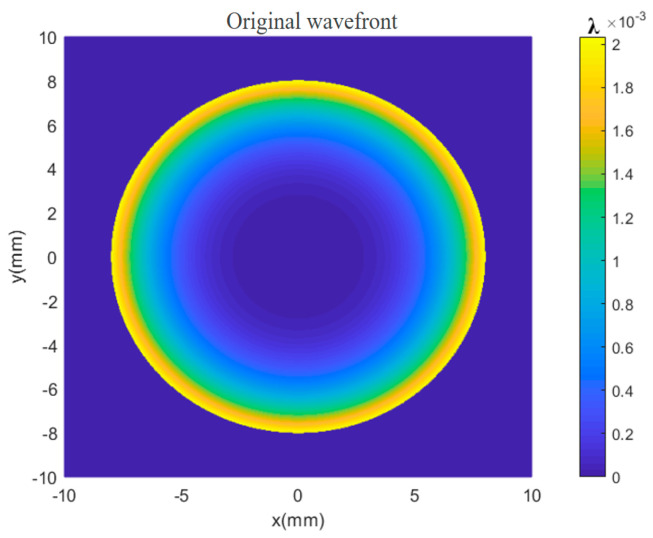
Original wavefront of the simulated aspherical surface.

The initial aspherical wavefront expression for the simulation is given by Equation (41), in which, the spherical parameters k=0 and c=1/100 are defined, respectively. The original wavefront was simulated using MATLAB as shown in Figure 8.

**Figure 9:**
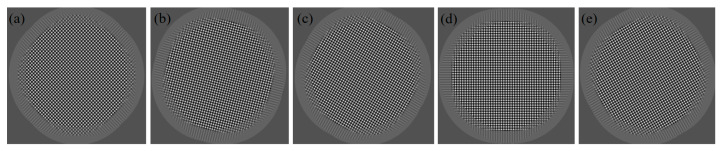
Simulation results of orthogonal lateral shearing interferograms: (**a**) 0° and 90°, (**b**) 15° and 105°, (**c**) 30° and 120°, (**d**) 45° and 135°, and (**e**) 60° and 150°.

The simulation results of orthogonal lateral shearing interferograms in any direction are shown in Figure 9. After performing a Fourier transform, spectral signals from different orthogonal directions are extracted. This allows the differential phases in these directions to be obtained, which are then used for wavefront reconstruction.

**Figure 10:**
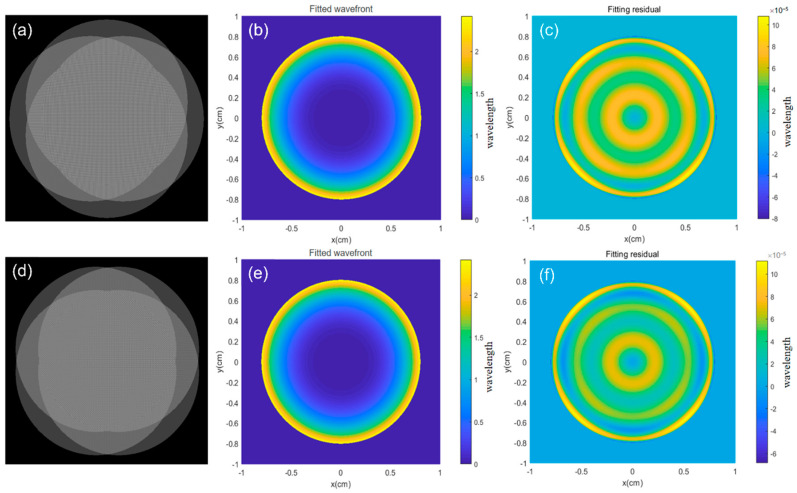
Simulation reconstruction results: (**a**) orthogonal lateral shearing interferogram at 0° and 90°, (**b**) fitted wavefront at 0° and 90°, (**c**) fitting residual at 0° and 90°, (**d**) orthogonal lateral shearing interferogram at 45° and 135°, and (**e**) fitted wavefront at 45° and 135°, (**f**) fitting residual at 45° and 135°.

The simulation reconstruction results are shown in Figure 10. The simulated orthogonal lateral shearing interferograms for two groups of arbitrary different directions used for wavefront reconstruction are also presented. The PV and RMS values of the reconstructed wavefront are shown in Table 1.

According to Table 1, the wavefront fitting data for orthogonal lateral shearing interferometry in two fixed directions were calculated. The ΔPV and ΔRMS of residual error showed only minimal change. By using a single interferogram, wavefront information in two orthogonal directions can be obtained. Multi-directional orthogonal lateral shearing interferometry increases the number of phase points, providing a more complete spatial height distribution. The shearing wavefront data from different shear directions are averaged first, and then the wavefront coefficients are solved and averaged again, effectively reducing the impact of system random errors. The differential Zernike polynomial coefficients, obtained by averaging multiple orthogonal directional wavefront combinations, facilitate high-resolution wavefront reconstruction. This data confirms the reliability and feasibility of multi-directional orthogonal lateral shearing interferometry for wavefront reconstruction, as demonstrated in the simulation study.

#### 3.1.2. Wavefront Deviation of the Aspherical Surface

**Figure 11:**
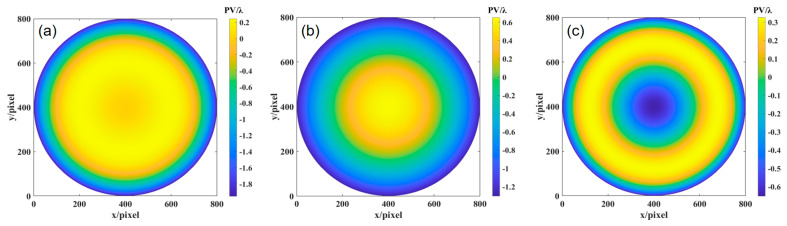
OPD, defocus, and wavefront deviation obtained through simulation fitting: (**a**) OPD obtained by ray tracing, (**b**) defocus error, and (**c**) wavefront deviation of the ideal aspherical surface relative to the optimal spherical surface.

In the investigation of aspherical wavefront, simulation calculations were performed with *k* = −1, *R* = 606, and aperture *D* = 90. These parameters yield the wavefront deviation between the ideal aspherical surface and the optimal spherical surface. According to Equation (31), the radius of the optimal comparison spherical surface is 606.845 mm. Using the ray-tracing model, the *OPD* was fitted with MATLAB, as shown in Figure 11a, where the PV value is 2.086λ and the RMS value is 0.608λ. The defocus error in Figure 11b has a PV value of 1.853λ and RMS value of 0.534λ. The wavefront deviation, obtained by subtracting the two, is shown in Figure 11c, with a PV value of 0.998λ and RMS value of 0.275λ.

**Figure 12:**
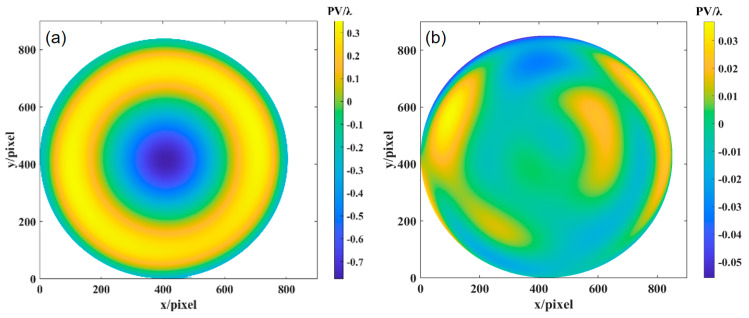
(**a**) Wavefront deviation of the paraboloid surface relative to the optimal spherical surface: (**b**) deviation of the aspherical surface relative to the design value.

Using the wavefront reconstruction method of multi-directional orthogonal lateral shearing interferometry, the Zernike coefficients obtained from differential information in different directions are averaged. These coefficients are then used to generate the final reconstructed wavefront by eliminating the translation, tilt, and defocus terms. The deviation of the tested paraboloid surface relative to the optimal spherical surface is then determined. The final wavefront deviation results are shown in Figure 12a, with a PV of 1.124λ and an RMS of 0.276λ.

To calculate the machining error of the aspherical surface relative to the design value, the measured value is compared with the design value. In the previous study, the ray-tracing method was used to calculate the *OPD*, and the deviation value of the ideal paraboloid surface relative to the optimal spherical surface was obtained through the MATLAB simulation, as shown in Figure 11c. By subtracting the two values, the deviation of the paraboloid surface relative to the ideal paraboloid surface can be determined. Figure 12b shows the wavefront deviation of the paraboloid surface obtained by orthogonal lateral shearing interferometry, with a PV of 0.097λ and an RMS of 0.026λ.

### 3.2. Experimental Result

#### 3.2.1. Testing Results of the Aspherical Surface with 4 μm Asphericity

**Figure 13:**
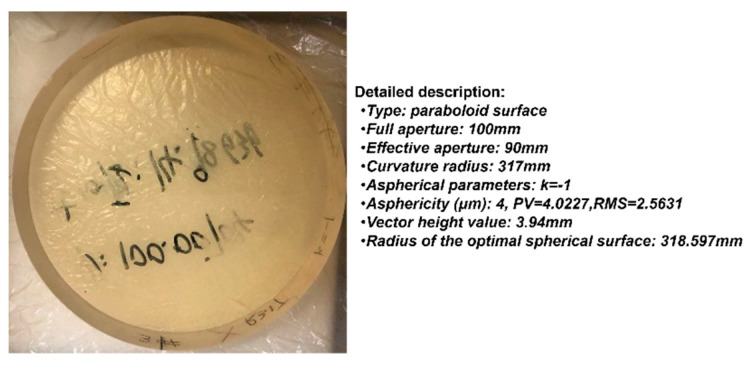
The aspherical mirror under test with 4 μm asphericity.

**Figure 14:**
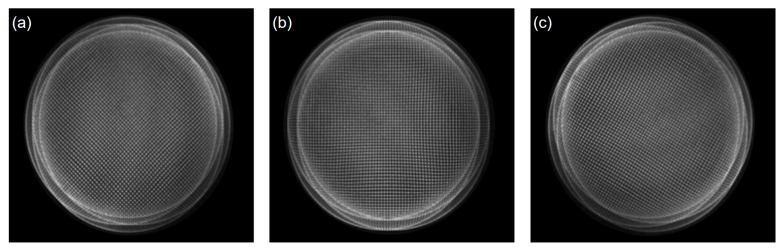
Orthogonal lateral shearing interferograms obtained from experiments in different directions: (**a**) 0°, (**b**) 45°, and (**c**) 120°.

The aspherical optical element was tested using a paraboloid mirror with a diameter of 100 mm, a curvature radius of 317 mm, and an asphericity of 4 μm. The tested aspherical mirror is shown in Figure 13. By performing rotational motion at multiple angles using the orthogonal shear module, multi-directional orthogonal lateral shearing interferograms were accurately captured, as shown in Figure 14.

**Figure 15:**
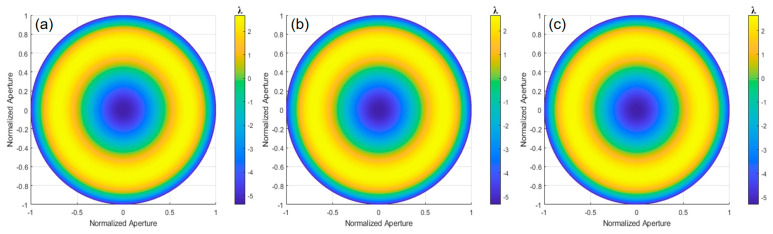
Wavefront reconstruction results from the experiment in orthogonal shear directions: (**a**) 0°; (**b**) 0° and 45°; and (**c**) 0°, 45°, and 120°.

The differential phase distribution is calculated through Fourier-transform phase extraction from Figure 14. A matrix relationship model is established between the differential phase in different orthogonal directions and the differential Zernike coefficients. The Zernike polynomial coefficients are obtained using the least squares method, and the Zernike polynomial surface is fitted to reconstruct the wavefront deviation of the paraboloid surface under test relative to the optimal spherical surface, as shown in Figure 15. The results of orthogonal shear wavefront reconstruction in different directions are shown in Table 2, with an average PV value of 7.9720λ and an average RMS value of 2.3652λ.

**Figure 16:**
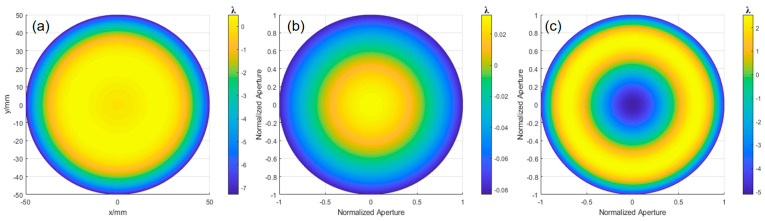
OPD, defocus, and surface deviation obtained through fitting: (**a**) OPD obtained by ray tracing, (**b**) defocus error, and (**c**) wavefront deviation of the ideal aspherical surface relative to the optimal spherical surface.

The experimental results were obtained based on the theoretical analysis of aspherical surface testing. The surface error of the experimental aspherical surface (with *k* = −1, *R* = 317 mm, and aperture *D* = 100 mm) was calculated, which reveals the wavefront deviation between the ideal aspherical surface and the optimal spherical surface. According to Equation (31), the radius of the optimal comparison spherical surface was calculated to be 318.597 mm. Using the ray-tracing model described earlier, the OPD is fitted with MATLAB, as shown in Figure 16a, yielding a PV value of 7.7950λ and an RMS value of 2.2967λ. The defocus error is shown in Figure 16b, with a PV value of 0.1155λ and an RMS value of 0.0334λ. Subtracting the two results gives the wavefront deviation shown in Figure 16c, with a PV value of 7.6654λ and an RMS value of 2.2794λ.

**Figure 17:**
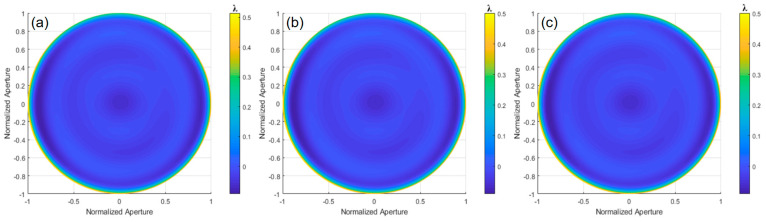
The wavefront deviation of the aspherical surface relative to the design value: (**a**) 0°, (**b**) 0° and 45°, and (**c**) 0°, 45°, and 120°.

Using the ray-tracing method, the *OPD* was calculated, and the deviation of the ideal paraboloid surface relative to the optimal spherical surface was obtained through the MATLAB simulation, as shown in Figure 16c. By subtracting the experimental results from the simulation results, the deviation of the paraboloid surface under test relative to the ideal paraboloid surface can be determined. Figure 17 shows the wavefront deviation of the paraboloid surface relative to the design value, measured by orthogonal lateral shearing interferometry. In the 0° direction, the wavefront deviation is PV: 0.6075λ and RMS: 0.0543λ. In the 0° and 45° directions, the wavefront deviation is PV: 0.6069λ and RMS: 0.0543λ. In the 0°, 45°, and 120° directions, the wavefront deviation is PV: 0.6065λ and RMS: 0.0539λ.

#### 3.2.2. Testing Results of the Aspherical Surface with Asphericity Greater than 10 μm

**Figure 18:**
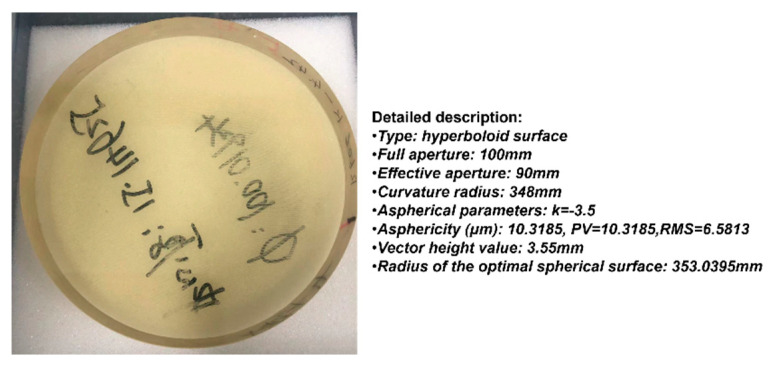
The aspherical mirror under test with 10.3185 μm asphericity.

**Figure 19:**
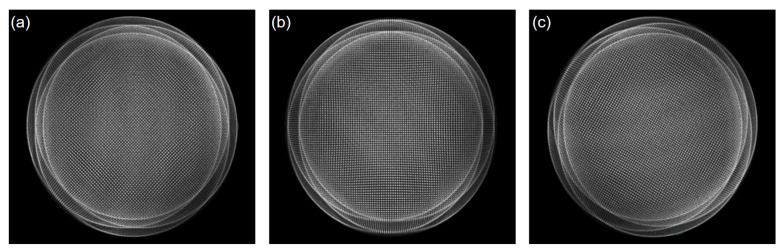
Orthogonal lateral shearing interferograms obtained from experiments in different directions: (**a**) 0°, (**b**) 45°, and (**c**) 120°.

To further verify that the multi-directional orthogonal lateral shearing interferometry method can be used for testing high-gradient aspherical surfaces, this section focuses on the measurement of another aspherical optical element: a hyperboloidal mirror with an aperture of 100 mm, a curvature radius of 348 mm, *k* = −3.5, and an asphericity of 10.3185 μm. The aspherical mirror under the test is shown in Figure 18, and its multi-directional orthogonal lateral shearing interferometry fringe patterns are shown in Figure 19.

**Figure 20:**
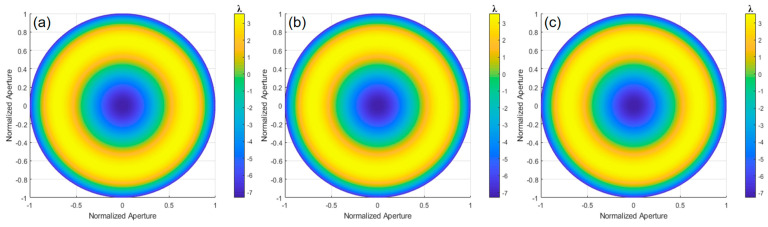
Wavefront reconstruction results of the experiment for orthogonal shear directions: (**a**) 0°, (**b**) 0° and 45°, and (**c**) 0°, 45°, and 120°.

The differential phase distribution was calculated by performing Fourier-transform phase extraction from Figure 19. After wavefront reconstruction, the deviation of the paraboloid surface under test relative to the optimal spherical surface is shown in Figure 20. The results of orthogonal shearing wavefront reconstruction in different directions are summarized in Table 3, with an average PV value of 10.8153λ and an average RMS value of 3.1729λ.

**Figure 21:**
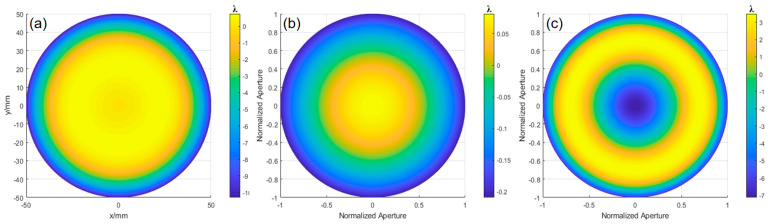
OPD, defocus, and surface deviation obtained through fitting: (**a**) OPD obtained by ray tracing, (**b**) defocus error, and (**c**) wavefront deviation of the ideal aspherical surface relative to the optimal spherical surface.

Based on the theory of aspherical surface measurement, the surface error of the experimentally tested aspherical mirror (with *k* = −3.5, *R* = 348 mm, and aperture *D* = 100 mm) is calculated, revealing the wavefront deviation between the ideal aspherical surface and the optimal spherical surface. Using Equation (31), the radius of the optimal comparison spherical surface is calculated to be 353.0395 mm. Then, applying the ray-tracing model described earlier, the *OPD* is fitted using MATLAB, as shown in Figure 21a, with a PV value of 11.0348λ and an RMS value of 3.2669λ. The defocus error, shown in Figure 21b, has a PV value of 0.2901λ and an RMS value of 0.0837λ. Subtracting these results gives the wavefront deviation shown in Figure 21c, with a PV value of 10.6157λ and an RMS value of 3.1184λ.

**Figure 22:**
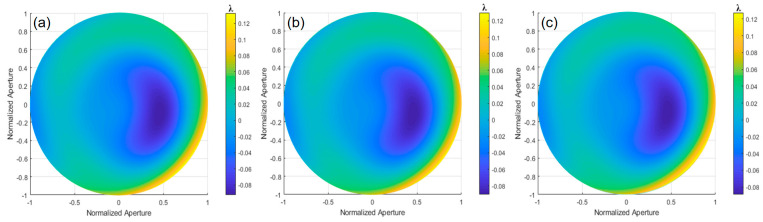
The wavefront deviation of the aspherical surface relative to the design value: (**a**) 0°, (**b**) 0° and 45°, and (**c**) 0°, 45°, and 120°.

Using the ray-tracing method, the *OPD* was calculated, and the deviation value of the ideal paraboloid surface relative to the optimal spherical surface was obtained through the MATLAB simulation, as shown in Figure 21c. By subtracting values obtained from between experimental and simulation results, the deviation of the tested paraboloid surface relative to the ideal paraboloid surface can be determined. Figure 22 shows the wavefront deviation of the paraboloid surface relative to the design value, measured by orthogonal lateral shearing interferometry. In the 0° direction, the wavefront deviation has a PV of 0.2247λ and an RMS of 0.0471λ. In the 0° and 45° directions, the PV is 0.2204λ and the RMS is 0.0457λ. In the 0°, 45°, and 120° directions, the PV is 0.2163λ and the RMS is 0.0444λ.

## 4. Discussion

### 4.1. Comparison with Existing Methods

**Figure 23:**
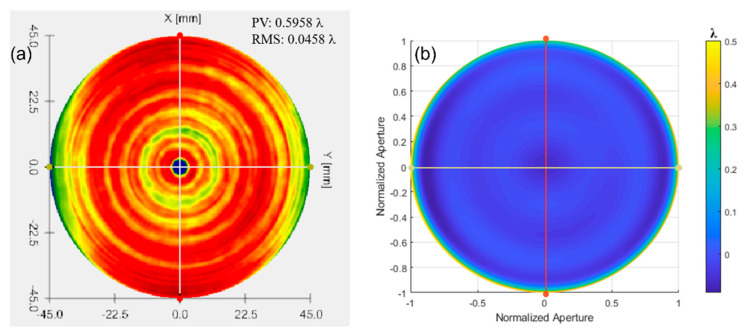
The wavefront deviation of the paraboloid surface relative to the design value: (**a**) measurement results from the Luphoscan, and (**b**) test results from the multi-directional orthogonal lateral shearing interferometry.

**Figure 24:**
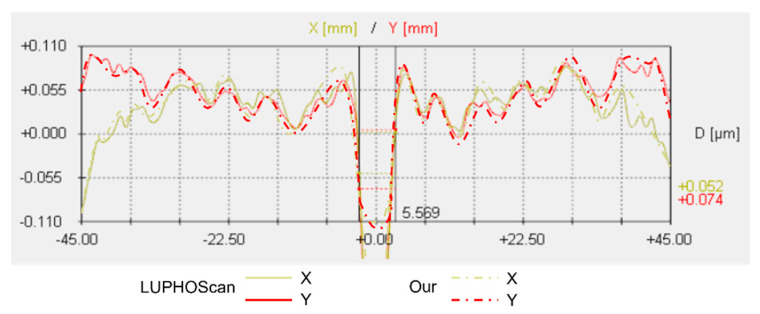
Profilograms were obtained from the arrays of digital data for an aspherical surface with an asphericity of 4 μm.

The Luphoscan, a non-contact 3D optical surface profilometer with the highest testing accuracy, was used to scan the paraboloid surface. The deviation of the surface under test relative to the ideal paraboloid was determined based on the measurements of an aspherical surface with a 4 μm asphericity. The measured result from the Luphoscan is shown in Figure 23a, while the testing result from multi-directional orthogonal lateral shearing interferometry is shown in Figure 23b. Profilograms were generated from the digital data arrays, as shown in Figure 24.

By comparing the scanning measurement results of the Luphoscan profilometer with those of the multi-directional orthogonal lateral shearing interferometry, it is found that the wavefront distribution of the paraboloid surface under test is consistent with the design value. Additionally, the data in Table 4 show that the wavefront deviation in PV values between the two methods is 0.0117λ, and the deviation in RMS values is 0.0085λ. Therefore, the RMS of wavefront deviation is very small, with a relative error is better than λ/100. These experimental results validate the effectiveness of the multi-directional orthogonal lateral shearing interferometry system for testing aspherical surfaces.

**Figure 25:**
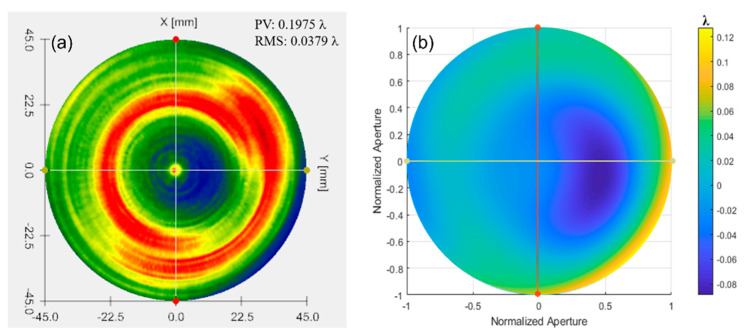
The wavefront deviation of the paraboloid surface relative to the design value: (**a**) measurement results from the Luphoscan, and (**b**) test results from the multi-directional orthogonal lateral shearing interferometry.

**Figure 26:**
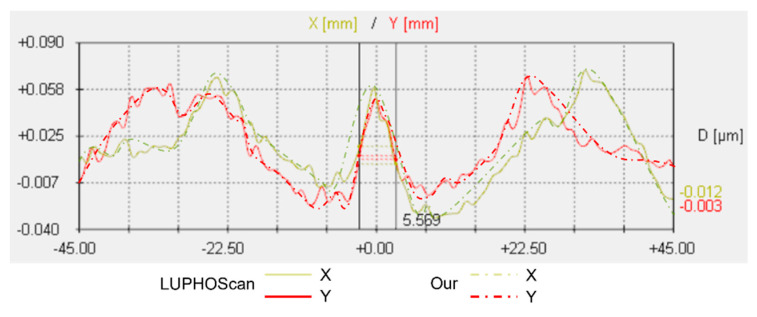
Profilograms were obtained from the arrays of digital data for an aspherical surface with an asphericity of 10.3185 μm.

According to the testing results of an aspherical surface with an asphericity of 10.3185 μm, the Luphoscan profilometer was used to perform probe scanning measurements on the aspherical surface under test, and measure the wavefront deviation relative to the ideal aspherical surface. The results from the Luphoscan profilometer are shown in Figure 25a, while the results from the multi-directional orthogonal lateral shearing interferometry are shown in Figure 25b. The profilograms, obtained from the digital data arrays, are shown in Figure 26. In our experiments, the same working wavelength and coordinate grid were used. After precise calibration, consistent images were obtained, ensuring reliable measurement results.

As shown in Figure 25 and Figure 26, the scanning measurement results from the Luphoscan profilometer are consistent with the test results obtained using the multi-directional orthogonal lateral shearing interferometry. Furthermore, the wavefront distribution of the aspherical surface under test aligns closely with the design specifications. The measurement comparison results are presented in Table 5, which show that the wavefront deviation in PV values between the two methods is 0.0229λ, and the deviation in RMS values is 0.0078λ. Therefore, the RMS wavefront deviation is minimal, with a relative error is better than λ/100. These experimental results strongly validate the effectiveness of the multi-directional orthogonal lateral shearing interferometry system for testing aspherical surfaces with an asphericity greater than 10 μm.

**Figure 27:**
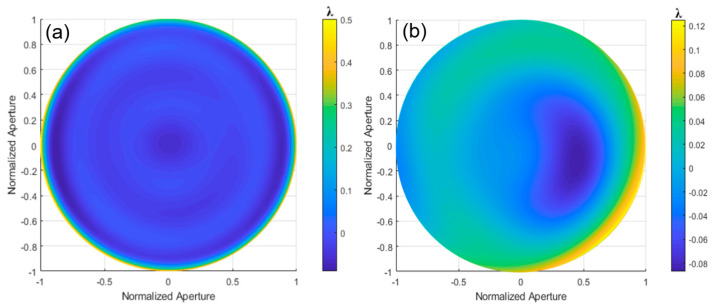
The wavefront deviation for the algorithms designed to improve accuracy is shown for aspherical surfaces with asphericities (**a**) 4 μm and (**b**) 10.3185 μm.

To improve the measurement accuracy by fitting large amounts of data and simplifying the analysis of complex surface geometries, the integral minimization method and multi-segment stitching are used for aspherical surface testing and wavefront reconstruction. The integration minimization method [21], based on the least squares principle, aims to find a wavefront expression that best fits the data by minimizing the difference between the measured wavefront slopes and the theoretically calculated slopes. The result of this reconstruction is shown in Figure 27a. Factors such as data accuracy, choice of polynomial basis functions, and calculation stability can affect wavefront reconstruction quality.

The multi-segment stitching method decomposes the complex aspheric wavefront into several simpler sub-wavefronts [22], which are obtained through interferometry and other techniques. The accurate measurement and positioning of the sub-wavefronts are required during the stitching process. However, the measurement equipment accuracy and environmental factors (e.g., temperature and vibration) can introduce errors, affecting the stitching accuracy. The reconstruction result is shown in Figure 27b. A comparison with multi-directional orthogonal lateral shearing interferometry is shown in Table 6.

The surface distribution of the measured aspherical is consistent with the design value when compared to the LuphoScan results. According to the data in Table 6, the PV deviation between the two methods is 0.0148λ, and the RMS deviation is 0.0053λ. The RMS deviation is very small, with a relative error precision better than λ/180, and the relative error has decreased by 32.05%. The experimental results confirm the effectiveness and accuracy of the multi-directional orthogonal lateral shearing interferometry system for aspherical surface measurement.

### 4.2. Retrace Error Implications

Aspherical surface wavefront testing using multi-directional orthogonal lateral shearing interferometry is with a partial compensation non-null measurement method. The retrace error in the system occurs because the incoming and reflected light rays follow different paths. The wavefront distribution obtained from the interferogram does not directly represent the aspherical surface; instead, it reflects both the retrace error and the surface’s wavefront after aliasing. To accurately reconstruct the surface’s wavefront from non-null interferograms, a retrace error correction algorithm is needed.

The impact of retrace error on wavefront aberrations shows that, to correct the error caused by asphericity, only the retrace error from the nominal aspherical surface should be treated as the system’s inherent error. This error can be accurately calculated using ray tracing. Additionally, the iterative reverse optimization (IRO) method, using multiple ray-tracing steps, is used to correct the retrace error in non-null aspherical interferometry testing.

Assuming the actual wavefront error $W_{s}$ of the aspherical surface and the wavefront error ${W^{\prime }}_{s}$ in the model, since the polished surface of the aspherical surface primarily exhibits smooth and gradual variations at medium and low frequencies, it can be represented by a finite series of Zernike polynomials as follows:

(42) $$W_{s}=\sum_{j=1}^{N}A_{j}Z_{j},\;{W^{\prime }}_{s}=\sum_{j=1}^{N}C_{j}Z_{j}$$

In this expression, $A_{j}$ and $C_{j}$ are the coefficients of the Zernike polynomial $Z_{j}$ corresponding to $W_{s}$ and ${W^{\prime }}_{s}$, respectively, and *N* is the number of polynomial terms.

Once the system’s structural parameters and aspherical parameters are identified, the wavefront distribution on the image plane is uniquely determined by the aspherical surface. Since the model is based entirely on the actual testing system, when the wavefront ${W^{\prime }}_{t}$ at the image plane in the model matches the reconstructed wavefront $W_{t}$ in the actual testing system, the aspherical surface error in the model corresponds to the actual aspherical surface. Therefore, we have the following:

(43) $$W_{t}=\sum_{j=1}^{N}B_{j}Z_{j},\;{W^{\prime }}_{t}=\sum_{j=1}^{N}{B^{\prime }}_{j}Z_{j}$$

In this expression, $B_{j}$ and ${B^{\prime }}_{j}$ are the coefficients of the Zernike polynomial $Z_{j}$ corresponding to $W_{t}$ and ${W^{\prime }}_{t}$, respectively.

The IRO retrace error correction algorithm is detailed in Ref. [23]. By modeling the optical path in Optic Studio ZEMAX based on actual test data, the Zernike polynomial coefficient $B_{j}$ of the reconstructed wavefront $W_{t}$ in the testing system is set as the optimization objective. The aspherical surface error ${W^{\prime }}_{s}$ is set as the optimization variable, and the coefficients $C_{j}$ are iteratively optimized based on the objective function until $W_{t}$ and ${W^{\prime }}_{t}$ are less than the threshold *ε*. The optimal aspherical surface coefficient $C_{j}^{o}$ can then be obtained. The target function is as follows:

(44) $$O(C_{j}^{o})=\min[{\left(W_{t}-{W^{\prime }}_{t}\right)}^{2}]=\min\left[\sum_{j=1}^{N}\omega_{j}^{2}{\left(B_{j}-{B^{\prime }}_{j}\right)}^{2}\right]<\varepsilon$$

In this expression, $\omega_{j}^{2}$ represents the optimization weight. After obtaining the optimal surface coefficient, the aspherical surface error can be determined through Zernike polynomial fitting.

**Figure 28:**
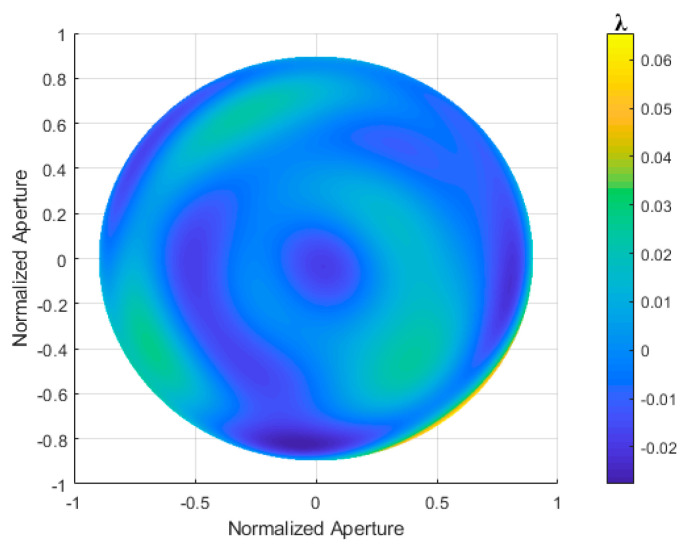
Original wavefront error of the aspherical surface is attached.

**Figure 29:**
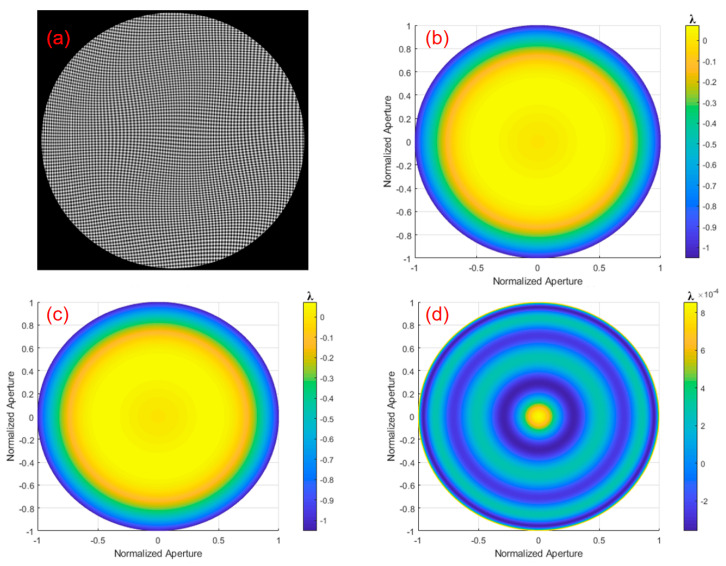
Calculation results for the simulated aspherical surface are shown as follows: (**a**) simulated interferogram, (**b**) original wavefront, (**c**) reconstructed wavefront, and (**d**) residual error.

To verify the accuracy of the aspherical surface testing system with retrace error correction, a simulated light source with a wavelength of 632.8 nm was used. The aspherical surface has the following parameters: cone constant *k* = −1, aperture *D* = 90 mm, curvature radius of the vertex spherical surface *R* = 606 mm, a high-order coefficient $A_{i}$ = 0 for the concave paraboloid surface, and an axial distance *d* = 606.8354 mm. The aspherical surface has an additional surface error with a PV value of 0.0973λ and an RMS value of 0.0267λ, which represents the original wavefront error *W_s_* of the aspherical surface, as shown in Figure 28. The calculation results for the simulated aspherical surface are shown in Figure 29.

The orthogonal lateral shearing interference pattern, obtained through simulation, is shown in Figure 29a, and the original wavefront from ray tracing is shown in Figure 29b. The simulation interferogram was demodulated using the wavefront reconstruction algorithm based on a multi-directional orthogonal differential Zernike polynomial, and the reconstructed wavefront is shown in Figure 29c. The residual error is shown in Figure 29d.

Table 7 presents the simulation results of wavefront reconstruction for an aspherical surface, with a residual error of 0.0012λ (PV value) and 2.3419 × 10^−4^λ (RMS value). Table 8 shows the simulation results of wavefront reconstruction for the aspherical surface before and after error correction using the IRO algorithm. The residual errors, obtained by subtracting the two surfaces, are 0.0092λ (PV value) and 0.0019λ (RMS value). These results demonstrate that the orthogonal lateral shearing interferometry system for aspherical surface testing has a high theoretical accuracy.

### 4.3. Wavefront Error Introduced by Two PBDs

We have also analyzed the influence of the orthogonal error based on DBCs-BD and the phase retrieval error when there is shear displacement when a tilted incident light strikes the DBCs-BD. Our investigation established that the correction range for the orthogonal angle error of the PBD is within −0.5° to 0.5°, with a maximum PV error of 0.0012 λ and a maximum RMS error of 1.3789 × 10^−4^ λ in wavefront reconstruction. Additionally, when the testing light tilts within the range of −0.4° to 0.4°, correcting the shear distance used for wavefront reconstruction yields high-precision results. For more details, please refer to the latest reference [24,25].

### 4.4. Further Consideration of Orthogonal Lateral Shearing Interferometry

As shown in Figure 2(a2,b2), the orthogonal lateral shear interferometry method is effective for representing aspherical wavefronts, but its success depends on the experimental design and data processing. For high-precision aspherical wavefront measurements, additional techniques may be required to enhance accuracy and reliability. Future work should focus on developing more efficient data-processing algorithms, optimizing experimental designs, and addressing the challenges posed by complex wavefront shapes, especially in high-precision measurements. Further consideration is needed in the following areas:

- Measurement accuracy and resolution: Aspherical wavefronts are more complex than spherical ones, often involving higher-order curvatures or intricate surface variations. When using orthogonal lateral shearing interferometry to measure these wavefronts, accuracy is crucial. While four-wave interferometry provides more detailed wavefront data than two-wave interferometry, factors like light source coherence and interferometer alignment can affect the accuracy of wavefront reconstruction. Therefore, it is important to evaluate whether this method can fully capture the high-frequency features of a complex aspherical wavefront or if distortion may occur.
- Complexity of interference pattern analysis: Aspherical wavefronts are often complex and may require advanced mathematical models for accurate reconstruction. It is still unclear whether orthogonal lateral shearing interferometry can effectively handle this complexity, especially for large-scale or high-precision measurements. More research is needed to explore whether advanced algorithms or different interference modes could better describe these wavefronts.
- Data processing and algorithm efficiency: Orthogonal lateral shearing interferometry requires processing large amounts of data from different angles, which poses challenges in data fitting and analysis. For higher-order aspherical wavefronts, the computational demands can be significant. Balancing the computational complexity with practical measurement needs is crucial. It is important to evaluate the strengths and weaknesses of current wavefront reconstruction algorithms and explore more efficient or accurate alternatives.
- Experimental design and error analysis: Effective experimental design and error control are crucial for accurate measurements. Environmental factors, such as vibrations and temperature changes, can affect the interference fringes in orthogonal lateral shearing interferometry. Therefore, it is important to model these errors and optimize algorithms to minimize their impact. Assessing error sources and using techniques like temperature control, mechanical stability, and data post-processing can help reduce errors and improve measurement reliability.

## 5. Conclusions

This paper presents an aspherical wavefront testing method based on multi-directional orthogonal lateral shearing interferometry, overcoming the limited phase sampling in traditional interferometers. Through theoretical analysis, simulations, and experimental validation, the method demonstrates high accuracy and effectiveness. The methodology of multi-directional orthogonal lateral shearing interferometry is described, and the theoretical foundations for testing aspherical surfaces are explored. The experimental setup is outlined, followed by results from simulations and experiments on aspherical surfaces with asphericities of 4 μm and 10.3185 μm. The measurement results were compared with those from the Luphoscan profilometer, showing a relative RMS accuracy better than 0.01λ. The experiment confirms the method’s effectiveness in testing aspherical wavefronts with asphericities greater than 10 μm. The discussion covers retrace errors and the numerical results of the error correction algorithm, further confirming the method’s feasibility and accuracy. Future improvements, such as incorporating polarization phase-shifting and Fourier reconstruction, could enhance testing accuracy.

## Figures and Tables

**Table 1:** PV and RMS values of the simulated reconstruction wavefronts.

Fitting Results	Original Wavefront	Wavefront Data of Orthogonal Lateral Shearing Interferometry (λ = 632.8 nm)		
		0°and 90°	0°, 90°, 45°, and 135°	Average Coefficient
PV (λ)	3.1222	3.1223	3.1322	3.1322
RMS (λ)	1.3211	1.3211	1.3211	1.3211
ΔPV (λ)	0	3.6391 × 10^−5^	2.8714 × 10^−5^	2.8654 × 10^−5^
ΔRMS (λ)	0	1.6764 × 10^−5^	9.8959 × 10^−6^	9.8873 × 10^−6^

**Table 2:** Reconstruction data of the orthogonal difference wavefront in different directions (λ = 632.8 nm).

Fitting Results	0° and 45°	0° and 120°	45° and 120°	0°, 45°, and 120°	Average Coefficients
PV (λ)	7.9848	7.7962	7.9856	7.9834	7.9720
RMS (λ)	2.3744	2.3748	2.3746	2.3721	2.3652

**Table 3:** Reconstruction data of the orthogonal differential wavefront in different directions (λ = 632.8 nm).

Fitting Results	0° and 45°	0° and 120°	45° and 120°	0°, 45°, and 120°	Average Coefficients
PV (λ)	10.9190	10.8879	10.8569	10.8262	10.8153
RMS (λ)	3.2075	3.1984	3.1893	3.1802	3.1729

**Table 4:** Comparison of the experimental test results with Luphoscan measurement results (λ = 632.8 nm).

Measurement Method	PV(λ)	RMS(λ)
Multi-directional orthogonal lateral shearing interferometry	0.6075	0.0543
Luphoscan profilometer scanning	0.5958	0.0458
The maximum differential value between them	0.0117	0.0085

**Table 5:** Comparison of the experimental test results with Luphoscan measurement results (λ = 632.8 nm).

Measurement Method	PV(λ)	RMS(λ)
Multi-directional orthogonal lateral shearing interferometry	0.2204	0.0457
Luphoscan profilometer scanning	0.1975	0.0379
The maximum differential value between them	0.0229	0.0078

**Table 6:** Comparison of the experimental test results with Luphoscan measurements (λ = 632.8 nm).

Measurement Method	PV(λ)	RMS(λ)
The algorithms designed to improve accuracy	0.2123	0.0432
Luphoscan profilometer scanning	0.1975	0.0379
The maximum differential value between them	0.0148	0.0053

**Table 7:** Simulation results of wavefront reconstruction for an aspherical surface (λ = 632.8 nm).

Reconstruction Results	Original Wavefront	Reconstruction Wavefront	Residual Error
PV (λ)	1.1247	1.1259	0.0012
RMS (λ)	0.3314	0.3316	2.3419 × 10^−4^

**Table 8:** Simulation results of reconstruction deviation with retrace error correction (λ = 632.8 nm).

Reconstruction Results	Original Wavefront	Reconstruction Wavefront	Residual Error
PV (λ)	0.0973	0.0964	0.0092
RMS (λ)	0.0267	0.0248	0.0019

## Data Availability

No data was generated or analyzed in the current study.
